# Phytochemical Study and Antibacterial and Antibiotic Modulation Activity of *Punica granatum* (Pomegranate) Leaves

**DOI:** 10.1155/2020/8271203

**Published:** 2020-03-26

**Authors:** Amine Trabelsi, Mohamed Amine El Kaibi, Aïmen Abbassi, Amira Horchani, Leila Chekir-Ghedira, Kamel Ghedira

**Affiliations:** ^1^Pharmacognosy Laboratory, Faculty of Pharmacy, University of Monastir, Avicenna Street, Monastir 5000, Tunisia; ^2^Research Unit of Bioactive and Natural Substances and Biotechnology UR17 ES49, Faculty of Dental Medicine, University of Monastir, Avicenna Street, Monastir 5000, Tunisia; ^3^Drug Development Laboratory (LR12ES09), Unit of Pharmacology, Faculty of Pharmacy, University of Monastir, Avicenna Street, Monastir 5000, Tunisia

## Abstract

This study aimed to determine phytochemical contents, antibacterial properties, and antibiotic modulating potential of *Punica granatum* leaf extracts: hexane, chloroform, ethyl acetate, ethanol, and aqueous extracts as well as an extract enriched with total oligomer flavonoids (TOFs). The TOF extract contained the highest value of phenols and flavonoids. Rutin, luteolin, gallic acid, and ellagic acid were determined by HPLC analysis of this extract. The antibacterial activity was assayed by the disc diffusion method and microdilution method against *Staphylococcus aureus* and *Escherichia coli* standard ATCC strains and clinical isolates resistant strains. The TOF extract was the most active against all tested strains. The checkerboard method was used for the determination of synergy between two antibiotics (amoxicillin and cefotaxime) and *P. granatum* leaf extracts. The best synergistic interaction was found with TOF extract combined with amoxicillin for penicillin-resistant *E. coli* and penicillin-resistant *S. aureus*. These results can be assigned to tannins, flavonoids, and phenolic acids found in *P. granatum* leaf extracts. Pomegranate leaf extracts or active compounds isolated from these extracts could be used to fight the emergence and spread of resistant bacterial strains.

## 1. Introduction

According to the 2014 report of the World Health Organization (WHO), antimicrobial resistance is an increasingly serious public health issue that is reaching alarming levels. The emergence of multidrug-resistant bacteria makes antimicrobial treatments ineffective, lengthens the duration of illness and the time of hospitalization, and increases the mortality rate [1]. One of the solutions is the combination of antibiotics and other agents in order to have a synergistic effect. Many projects aim at developing a new generation of phytopharmaceuticals which can be used alone or in combination with antibiotics. This new generation of phytopharmaceuticals could lend phytotherapy a new legitimacy and it can give the possibility of treating diseases classically treated with synthetic drugs [2].

*Punica granatum* L. commonly known as pomegranate is a small tree of the family of Lythraceae. It is native from Persia and has been cultivated extensively in the Mediterranean countries such as Tunisia, Turkey, Egypt, and Spain and to some extent also in California, China, Japan, and Russia [3]. Constituents of *P. granatum* have been involved in many different biological and pharmacological activities. It has been observed that the phenolics contained in pomegranate leaves predominantly contributed to their health benefits. These phenolics possess a strong binding ability to different molecular structures like proteins or glycoproteins that can antagonize bacterial resistance [2].

Hence, this study aimed to determine phytochemical contents, antibacterial properties, and antibiotic modulation potential of six different pomegranate leaf extracts.

## 2. Material and Methods

### 2.1. Plant Material

The leaves of *P. granatum* (Gabsi cultivar) were collected from the region of Gabes situated in the South of Tunisia, in September 2015. A voucher specimen (Herbarium 23/2015) has been kept in the laboratory of pharmacognosy (Faculty of Pharmacy of Monastir, Tunisia) for future reference. The leaves were shade dried, powdered, and stored in a tightly closed container for further use.

### 2.2. Preparation of Plant Extracts

Hexane, chloroform, ethyl acetate, and ethanol extracts were obtained by successive Soxhlet extraction (6 h). These four extracts, with increasing polarities, were concentrated to dryness and kept at 4°C.

The aqueous extract was prepared by a decoction of 50 g of plant material in 500 mL of boiling water for 20 minutes. After filtration, the extract was lyophilized and kept at 4°C.

The extract enriched with total oligomer flavonoids (TOFs) was obtained, as described by [4], by maceration in a mixture of acetone/water (2 V/1 V). After filtration, the acetone was evaporated under reduced pressure and the residual aqueous phase was saturated with NaCl. The formed precipitate overnight at 4°C was removed by filtration. Liquid-liquid extraction was carried out with ethyl acetate. The organic phase was concentrated under reduced pressure and then poured into an excess of chloroform. The resulting flocculate corresponds to the TOF.

For phytochemical study and antibacterial and antibiotic modulation assays, each extract was dissolved in the adequate solvent. The same solvent was used in the corresponding negative control.

### 2.3. Phytochemical Study

#### 2.3.1. Preliminary Phytochemical Screening

Extracts were screened for the presence of secondary metabolites such as tannins, flavonoids, saponins, cardiac glycosides, anthocyanins, and alkaloids. The secondary metabolites were detected qualitatively by the characteristic color changes occurring upon treatment with specific reagents [5].

#### 2.3.2. Determination of Total Contents of Phenols, Flavonoids, and Tannins

The total content of phenols of extracts from *P. granatum* was determined by the Folin-Ciocalteau method [6]. Indeed, to 100 *μ*L of a solution of extract, 2 mL of sodium carbonate (Na_2_ CO_3_) at 2% and 0.1 mL of Folin-Ciocalteu reagent at 50% were added. After incubation for 30 minutes at room temperature, a petroleum blue color indicates the presence of polyphenols which were measured at the wavelength of 720 nm. Gallic acid was used as a standard, and results were expressed as milligram of gallic acid equivalents (GAEs) per gram of dry mass (DM).

The content of flavonoids of each extract was determined by the aluminum chloride colorimetric method [7]. This method consists in adding 75 *μ*L of 5% NaNO_2_, 150 *μ*L of 10% AlCl_3_, and finally 500 *μ*L of 1 N NaOH to 250 *μ*L of a solution of leaf extract. The volume was adjusted to 2.5 mL and incubated for 5 minutes at room temperature. Absorbance was measured at 510 nm. Quercetin was used as a standard and results were expressed as milligram of quercetin equivalents (QEs) per gram of DM.

The content of tannins was determined by the Folin-Denis [8]. We added 10 *μ*L of Folin-Denis reagent to 50 *μ*L of extract, followed by 25 *μ*L of saturated sodium carbonate solution, which was then incubated at room temperature for 90 minutes. The absorbance was measured at 760 nm. Tannic acid was used as standard and results were expressed as milligram of tannic acid equivalents (TAEs) per 100 g of DM.

#### 2.3.3. HPLC Characterization of TOF Extraction

A high-performance liquid chromatographic method with gradient elution and diode-array detection was used to quantify some phenolic acids (gallic acid, ellagic acid) and flavonoids (rutin, luteolin) in TOF extract.

HPLC analyses were performed with Shimadzu LC-2030 3D Plus (Prominence-i) chromatograph. The mobile phase contains 1% aqueous acetic acid solution (solvent A) and acetonitrile (solvent B), the flow rate was adjusted to 0.7 mL/min, the column was thermostatically controlled at 28°C, and the injection volume was kept at 10 *μ*L. Gradient elution was performed by varying the proportion of solvent B to solvent A: from 10% to 40% B for 28 min, from 40 to 60% B for 39 min, and from 60 to 90% B for 50 min. The mobile phase composition was set back to the initial condition (solvent B: solvent A: 10 : 90) in 55 min and allowed to run for another 10 min, before the injection of another sample. Chromatographic separation was performed on a Chrom-CloneTM C18 column (5 *μ*m particle size, 250 × 4.6 mm), and the detection was conducted using a diode-array UV detector at 254 nm [9].

Standard solution of gallic acid, ellagic acid, rutin, and luteolin was prepared in methanol (0.1 mg/mL). The sample solution was prepared by dissolving 1 mg of TOF extract in 1 of mL methanol. Both the standard and sample solutions were filtered through a Whatman 0.45 *μ*m syringe filter. The responses were measured as peak areas versus concentration.

### 2.4. Antibacterial Activity

#### 2.4.1. Bacterial Strains

Antibacterial activity of *P. granatum* extracts was determined against Gram-positive bacteria (*Staphylococcus aureus*) and Gram-negative bacteria (*Escherichia coli*). Extracts were tested against bacterial strains obtained from the American Type Culture Collection (*S. aureus* ATCC 25923 and *E. coli* ATCC 25922) and clinical isolates strains obtained from the department of microbiology (University Hospital of Monastir) with resistance profile described in Table 1.

Antibiotic modulation activity was determined against resistant strains: penicillin-resistant *S. aureus*, methicillin-resistant*S. aureus*, penicillin-resistant *E. coli*, and multidrug-resistant *E. coli*.

#### 2.4.2. Disc Diffusion Method

The disc diffusion method was performed according to the method reported by the National Committee for Clinical Laboratory Standards (NCCLS). Sterile paper disks of 6 mm diameter were impregnated with different extracts and then air-dried. Each disk contains approximately 20 *μ*L of extract at the concentration of 5 mg/mL. Blank disks, impregnated with solvents, were used as a negative control. These paper discs were placed on Müller-Hinton agar inoculated with the tested bacteria (10^8^ CFU/mL). Following incubation at 37°C for 24 hours, diameters of inhibition zones were measured in millimeter scale.

#### 2.4.3. Microdilution Method

The microdilution method using 96-well microplates and 3-(4,5-dimethylthiazol-2-yl)-2,5-diphenyltetrazolium bromide (MTT) to indicate bacterial growth was used to determine the Minimal Inhibitory Concentration (MIC) [10, 11]. An inoculum of 100 *μ*L of each strain suspended in Müller-Hinton broth up to a final concentration of 10^6^ CFU/mL was used in 96-well microplates. Each well received 100 *μ*L of each extract solution. Using twofold serial dilutions, the final concentrations of the extracts varied from 5 to 0.04 mg/mL. After incubation at 37°C for 24 hours, 40 *μ*L of MTT at the concentration of 0.2 mg/mL was added to each well. After incubation for 30 minutes, MICs, which are the lowest concentrations required to inhibit growth, were recorded as the lowest concentrations required to inhibit the color change of the bacterial suspension to blue.

### 2.5. Antibiotic Modulation

The checkerboard method, which is commonly used for the determination of synergy between the antibiotics and natural antibacterials, was used for the antibiotic modulation assay [12]. This method is combined with a calculation of a fractional inhibitory concentration (FIC) index. To evaluate the synergy of our extracts with amoxicillin and cefotaxime, we started by determining the MIC of these antibiotics (MIC_ATB_) and the MIC of our extracts (MIC_Ext_) alone, and then we determined their new MIC after combination (MIC'_ATB_ and MIC'_Ext_). FIC must be determined to judge the interaction between the antibiotic and the natural extract:

FIC = FIC_A_ + FIC_B_ with FIC_A_ = MIC'_ATB_/MIC_ATB_ and FIC_B_ = MIC'_Ext_/MIC_Ext_

“Synergy” was defined by an FIC index ≤ 0.5. When the FIC index fell between 0.5 and 4.0, it indicated that there was “no interaction” between the agents. An FIC index = 4.0 would indicate that there was an “antagonism” between the two agents.

The microdilution method using tetrazolium salt [10] was used to determine the MIC of plant extracts and MIC of amoxicillin and cefotaxime. The initial concentrations of the agents used were determined according to their MIC value and then were serially diluted in twofold steps.

## 3. Results and Discussion

### 3.1. Phytochemical Study

In this study, six extracts were prepared from the powdered leaves of *P. granatum*. The yield of extracts depended on extraction solvents and extraction methods and varied from 0.5 to 20.07% (Table 2). The highest extraction yield was obtained with the aqueous extract and the lowest yield was obtained with the ethyl acetate extract.

The preliminary phytochemical study showed the presence of flavonoids and hydrolysable tannins in ethanol extract, aqueous extract, and TOF extract. Our results are comparable to the previous findings that identified phenolic acids, flavonoids, and hydrolysable tannins in *P. granatum* leaves [13–15]. But, some reports found, in addition to flavonoids and tannins, other secondary metabolites such as alkaloids [16] and anthocyanins [17].

Based on the results of the preliminary phytochemical study, the total contents of phenols, flavonoids, and tannins in leaf extracts of *P. granatum* were determined. These results are shown in Table 2. The TOF contained the highest value of phenols (382.27 ± 6.46 mg GAE/g DM) and flavonoids (492.88 ± 19.25 mg QE/g DM), followed by the aqueous extract (262.98 ± 5.11 mg GAE/g DM and 287.33 ± 15.55 mg QE/g DM, respectively). The aqueous extract showed the highest content of tannins (125.92 ± 3.9 mg TAE/100 g DM) followed by the ethanol extract (96.81 ± 2.27 mg TAE/100 g DM). Phenols, flavonoids, and tannins were absent in hexane extract. We also did not find flavonoids in chloroform extract. Polar extracts are richer in different secondary metabolites than nonpolar extracts. In fact, the phenolic nature of polyphenols such as flavonoids and tannins makes them relatively hydrophilic and highly soluble in water and polar organic solvents such as methanol, ethanol, and acetonitrile, or their mixture of water [18]. TOF contained the highest value of phenols and flavonoids but contained fewer tannins since it has been obtained using purification methods that removed tannins and enriched the extract with flavonoids. The total contents of phenols (410.85 ± 10.15 mg GAE/g DM) and flavonoids (286.88 ± 20.73 mg QE/g DM) in our study are greater than those reported by [17] which are 289.76 ± 1.55 mg GAE/g DM and 82.6 ± 2.67 QE/g DM, respectively, in extracts obtained by successive extraction using cold maceration method with solvents of increasing polarity (hexane, dichloromethane, ethyl acetate, ethanol, and methanol). Such a difference may be explained by the extraction method and the harvest time. Indeed, we prepared our extracts by successive extraction using the Soxhlet apparatus which gives better efficiency than cold maceration. Soxhlet method uses a fresh solvent that is always provided to the sample causing maximum analyte solubility [19]. Harvest time has an influence on the amount of phenols and flavonoids. Total levels of phenolics and flavonoids in pomegranate leaves are low in the early stages of leaf growth and then increased gradually until the end of September [16]. Regarding tannins assay, we found the highest values in polar extracts (aqueous extract, ethanol extract). Tannins are usually extracted from natural tissues by aqueous solutions of methanol, ethanol, or acetone, as well as ethyl acetate. Nonpolar organic solvents (hexane, chloroform, and dichloromethane) have low extraction strength for tannins [20].

**Figure 1:**
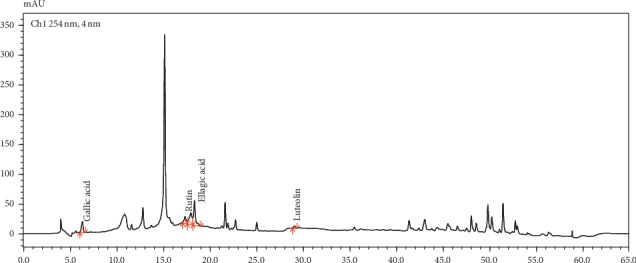
HPLC chromatogram of TOF extract of *Punica granatum* leaves.

Two flavonoids and two phenolic acids were determined by HPLC analysis of TOF extract (Figure 1). The flavonoids are rutin (3.56 ± 0.11 mg/g) and luteolin (0.81 ± 0.031 mg/g). The phenolic acids are gallic acid (9.65 ± 0.092 mg/g) and ellagic acid (8.56 ± 1.54 mg/g). The chromatogram also contained other unidentified peaks.

It has been reported that *P. granatum* leaf contains phenolic acids (gallic acid and ellagic acid), flavonoids (apigenin, luteolin, rutin, etc.), and hydrolysable tannins (punicafolin, corilagin, granatin, etc.) [13–15, 21].

### 3.2. Antibacterial Activity

The antibacterial activity of *P. granatum* extracts was assayed in vitro by agar disc diffusion method and microdilution method against Gram-positive and Gram-negative human pathogenic bacteria.  Table 3 summarizes the microbial growth inhibition of different extracts. The in vitro antibacterial study against the standard and resistant strains of *S. aureus* and *E. coli* showed that *P. granatum* leaf extracts exhibited various levels of antibacterial effect against all tested bacterial strains. Both techniques, disc diffusion and microdilution, have given consistent results. The inhibition zone diameters varied from 8 to 19 mm and MIC values varied from 0.625 to over 5 mg/mL. The TOF extract was the most active against all tested strains. In fact, this extract showed a remarkable activity against the Gram-positive and Gram-negative standard strains (MIC = 0.625 mg/mL) and the penicillin-resistant strains (MIC = 2.5 mg/mL). It also displayed an interesting activity against the methicillin-resistant *S. aureus* (MIC = 1.25 mg/mL) which is a major nosocomial pathogen worldwide and only some antibiotics such as vancomycin and teicoplanin are active against it [22]. The ethanol extract was active against all *S. aureus* strains and standard *E. coli* strains (MIC = 2.5 mg/mL). The aqueous extract was especially active against *E. coli* standard strain (MIC = 1.25 mg/mL) and *S. aureus* standard strain (MIC = 2.5 mg/mL).

This antimicrobial activity can be assigned to tannins and flavonoids found in *P. granatum* leaf extracts. These phytochemical groups are known to possess antimicrobial compounds [23, 24]. Their presence in the plant extracts could, therefore, justify the observed activity especially with the TOF extract, aqueous extract, and the ethanol extracts which are the richest of polyphenols. The antimicrobial effects of pomegranate leaves were previously studied by the disk diffusion method. Indeed, these studies showed that the methanolic extract exhibited the best inhibition against all tested strains (*Staphylococcus aureus, Bacillus cereus, Escherichia coli, Proteus mirabilis,* and *Salmonella typhi*) [25]. Methanolic leaf extract of *P. granatum* also showed antibacterial activity against the predominant bacterial isolates of septic wounds that are multidrug-resistant *P. aeruginosa, S. aureus, K. pneumoniae,* and *E. coli* [26]. The antimicrobial activity of ethyl acetate extract of pomegranate leaves was also studied by the microdilution method and showed MIC of 3.75 mg/mL for *P. aeruginosa*, MIC of 7.5 mg/mL for *E. coli*, and MIC of 15 mg/mL for *S. aureus* [27].

### 3.3. Antibiotic Modulation

The potential reduction of antibiotic resistance by *P. granatum* extracts was carried out by calculation of the FIC index shown in Table 4. The best synergistic interaction was found with TOF extract combined with amoxicillin for penicillin-resistant *E. coli* and penicillin-resistant *S. aureus* (FIC = 0.125). TOF extract showed also synergistic effect with cefotaxime for methicillin-resistant *S. aureus* (FIC = 0.5). The aqueous extract and ethanol extract were found to have a similar effect in reducing the resistance of penicillin-resistant *S. aureus* with amoxicillin (FIC = 0.25). Chloroform extract and ethyl acetate extract showed no synergistic effect with amoxicillin and cefotaxime for all tested resistant strains.

In the interaction study, polar extracts such as TOF, ethanol, and aqueous extracts were found to be highly effective especially against penicillin-resistant strains. These extracts contained the highest amount of phenolic compounds such as flavonoids. In fact, many studies showed that, in synergy with antibiotics, phenolic compounds such as flavonoids, tannins, and phenolic acids pose a promising alternative for therapeutic strategies against drug-resistant bacteria. The proposed mechanisms of synergy were the co-permeabilization of the bacterial membrane and the inhibition of penicillinase activity [28, 29]. The work [30] showed that pomegranate ethanol extract inhibited recombinant New Delhi metallo-*β*-lactamase 1 (NDM-1) activity and the IC_50_ value was 0.76 ng/*μ*L. The same extract caused an increase in NDM-1 *E. coli* cell permeability. In addition, methanol *P. granatum* leaf extract was able to produce a significant inhibition of biofilm formation for *L. monocytogenes* and *S. aureus* [31]. It should be noted that the formation of biofilm is an important mechanism of bacterial resistance. Finally, gallic acid, which is a phenolic acid present in TOF extract, showed a synergistic interaction with tetracycline by inhibiting the efflux pump in multidrug-resistant *E. coli* [32]. To our knowledge, no study in literature measured the antibiotic modulation potential of pomegranate leaf extracts with *β*-lactam antibiotics.

## 4. Conclusion

The phytochemical preliminary screening of *P. granatum* leaf extracts showed the presence of hydrolysable tannins and flavonoids. The quantitative analysis of total phenolics, flavonoids, and tannins carried out with spectrophotometric methods had confirmed these results particularly with TOF, aqueous, and ethanol extracts. The antibacterial study showed that the TOF extract was the most active extract against all tested strains. The most important result was the activity shown by the former extract against the methicillin-resistant *Staphylococcus aureus*. TOF extract also showed the best synergistic interaction with amoxicillin for penicillin-resistant *E. coli* and penicillin-resistant *S. aureus*. HPLC determination of flavonoids and phenolic acids in TOF extract showed the presence of rutin, luteolin, gallic acid, ellagic acid, and other undetermined substances.

In conclusion, pomegranate leaf extracts or active compounds isolated from those extracts could be used to improve human health especially the prevention and treatment of infectious diseases and to fight the emergence and spread of resistant bacterial strains. Nevertheless, further researches are still needed to evaluate the mechanisms of action and toxicity of those extract.

## Figures and Tables

**Table 1:** Phenotypic resistance profile of clinical isolates bacterial strains.

Bacterial strains	Clinical sample	Resistance to antibiotics	Phenotypic resistance profile
*Staphylococcus aureus*	Ascitic fluid	Penicillin G	Penicillin-resistant *S. aureus*
	Pus	Penicillin G	Methicillin-resistant *S. aureus*
		Oxacillin	
		Cefoxitin	

*Escherichia coli*	Urine	Amoxicillin	Penicillin-resistant *E. coli*
		Ticarcillin	
		Piperacillin	
		Amoxicillin + clavulanic acid	
		Ticarcillin + clavulanic acid	
	Urine	Amoxicillin	Multidrug-resistant *E. coli*
		Ticarcillin	
		Ceftazidime	
		Ticarcillin + clavulanic acid	
		Piperacillin	
		Cefotaxime	
		Cefalotin	
		Amoxicillin + clavulanic acid	
		Gentamycin	
		Tobramycin	
		Ciprofloxacin	
		Ofloxacin	
		Norfloxacin	
		Rifamycin	

**Table 2:** Extraction yields, total contents of phenols, flavonoids, and tannins of extracts from *P. granatum* leaves.

	Extracts					
	Hexane extract	Chloroform extract	Ethyl acetate extract	Ethanol extract	Aqueous extract	TOF extract
Extraction yield (%)	0.50	1.33	0.74	6.36	20.07	2.17
Phenols mg GAE/g DM	ND	49.24 ± 5.65	122.98 ± 1.27	238.63 ± 3.23	262.98 ± 5.11	382.27 ± 6.46
Flavonoids mg QE/g DM	ND	ND	55.11 ± 5.92	231.77 ± 14.81	287.33 ± 15.55	492.88 ± 19.25
Tannins mg TAE/100 g DM	ND	30.68 ± 0.91	56.13 ± 2.32	96.81 ± 2.27	125.92 ± 3.9	86.46 ± 2.1

ND: not detected.

**Table 3:** Antibacterial activity of *P. granatum* leaf extracts by microdilution method: Minimal Inhibitory Concentration was expressed in mg/mL (diameters of inhibition zones were measured in millimeter scale).

Extract	*Staphylococcus aureus*			*Escherichia coli*		
	Standard strain ATCC 25923	Penicillin-resistant strain	Methicillin-resistant strain	Standard strain ATCC 25922	Penicillin-resistant strain	Multidrug-resistant strain
Hexane extract	>5 (-)	>5 (-)	>5 (-)	>5 (-)	>5 (-)	>5 (-)
Chloroform extract	5 (-)	>5 (-)	>5 (-)	2.5 (-)	2.5 (-)	5 (-)
Ethyl acetate extract	5 (-)	>5 (-)	2.5 (8)	2.5 (8)	>5 (-)	5 (-)
Ethanol extract	2.5 (17)	2.5 (14)	2.5 (8)	2.5 (13)	5 (13)	5 (-)
Aqueous extract	2.5 (19)	5 (15)	5 (10)	1.25 (13)	5 (14)	5 (12)
TOF extract	0.625 (14)	2.5 (15)	1.25 (17)	0.625 (16)	2.5 (12)	5 (12)
Amoxicillin	0.001	0.064	0.128	0.001	0.256	0.512
Cefotaxime	0.001	0.002	0.128	0.001	0.001	0.512

**Table 4:** Antibiotic modulation activity of *P. granatum* leaf extracts by checkerboard method (FIC index).

Extract	*Staphylococcus aureus*				*Escherichia coli*			
	Penicillin-resistant strain		Methicillin-resistant strain		Penicillin-resistant strain		Multidrug-resistant strain	
	Amoxicillin	Cefotaxime	Amoxicillin	Cefotaxime	Amoxicillin	Cefotaxime	Amoxicillin	Cefotaxime
Chloroform extract	1	—	2	2	1	—	2	1
Ethyl acetate extract	2	—	2	2	1	—	2	1
Ethanol extract	0.25	—	2	1	2	—	2	1
Aqueous extract	0.25	—	2	1	0.5	—	2	0.5
TOF extract	0.125	—	2	0.5	0.125	—	2	0.5

## Data Availability

The data used to support the findings of this study are included within the article.
